# Assessment and Management of Children with Visual Impairment

**DOI:** 10.4103/0974-9233.53863

**Published:** 2009

**Authors:** Taha A. Labib, Mohamed A. El Sada, Boshra Mohamed, Neveen M. Sabra, Hanan M. Abdel Aleem

**Affiliations:** From the Faculty of Medicine, Cairo University, Egypt; 1 From the Memorial Institute of Ophthalmology, Giza, Egypt; 2 From the Research Institute of Ophthalmology, Giza, Egypt

**Keywords:** Low Vision, Visual Aids, Contrast Sensitivity, Visual Acuity

## Abstract

**Purpose:**

The aim of this work was to evaluate the role of low vision aids in improving visual performance and response in children with low vision.

**Study Design:**

Prospective clinical case series.

**Materials and Methods:**

This study was conducted on 50 patients that met the international criteria for a diagnosis of low vision. Their ages ranged from 5 to 15 years. Assessment of low vision included distance and near visual acuity assessment, color vision and contrast sensitivity function. Low vision aids were prescribed based on initial evaluation and the patient's visual needs. Patients were followed up for 1 year using the tests done at the initial examination and a visual function assessment questionnaire.

**Results:**

The duration of visual impairment ranged from 1 to 10 years, with mean duration ± SD being 4.6± 2.3299. The near visual acuities ranged from A10 to A20, with mean near acuity ± SD being A13.632 ± 3.17171. Far visual acuities ranged from 6/60 (0.06) to 6/24 (0.25), with mean far visual acuity ± SD being 0.122 ± 0.1191. All patients had impaired contrast sensitivity function as tested using the vision contrast testing system (VCTS) chart for all spatial frequencies. Distance and near vision aids were prescribed according to the visual acuity and the visual needs of every patient. All patients in the age group 5-7 years could be integrated in mainstream schools. The remaining patients that were already integrated in schools demonstrated greater independency regarding reading books and copying from blackboards.

**Conclusion:**

Our study confirmed that low vision aids could play an effective role in minimizing the impact of low vision and improving the visual performance of children with low vision, leading to maximizing their social and educational integration.

## INTRODUCTION

Low vision means visual abilities that are less than needed by the patient for the performance of their essential daily activities. In 1992, WHO defined a person with low vision as the one who has impairment of the visual function even after treatment and/ or standard refractive correction, and one who has a visual acuity of less than 6/18 to light perception, or a visual field of less than 10 degrees from the point of fixation, but the person uses or is potentially able to use vision for planning or execution of a task.^1^ It is a major handicap that has a great impact on educational as well as the social life of children and adolescents. To minimize the impact of this problem, early diagnosis, careful assessment and successful management of low vision is of great value since it can maximize the usefulness of any residual vision.

Socially, children with visual impairment have limitations in interacting with the environment, as they cannot see the facial expressions of parents and teachers; cannot perceive social behaviors; and sometimes, are unaware of the presence of others unless a sound is made.^2^ Psychologically, low vision has consequences that often lead children to become confused, fearful, anxious and depressed. Also denial, withdrawal and autism are common psychological problems that restrict the children from being socially as well as educationally integrated.^3^ In the educational environment, the major challenge facing the visually impaired students is the overwhelming mass of the visual materials to which they are continually exposed.^4^ Therefore, low vision has a wide-ranging impact on the lives of children, which should be accurately assessed and managed to lessen subsequent disability and handicap that limit the integration of the visually impaired patients into many of the daily life activities. Vision rehabilitation strategy is a multidisciplinary approach involving many services.^5^ Most of the causes of blindness and low vision are preventable, so screening of preschool and school children is very important to diagnose and manage any ocular pathology which may cause visual impairment, and to allow early implementation of vision rehabilitation programs, will help minimize the impact of visual impairment and maximize the efficacy of low vision aids.^6^

The aim of this work was to evaluate the role of low vision aids in improving visual performance and response in children with low vision.

## MATERIALS AND METHODS

Fifty patients, 27 males and 23 females, with ages ranging from 5 to 15 years that were diagnosed with low vision^1^ from various causes were included in the study. Patients in whom visual impairment was caused by curable causes were excluded from the study. Visual acuity for distance was measured for all patients using the Landolt's C chart, and the corresponding visual acuity was recorded. Near visual acuity was measured for all patients using Keeler's reading chart; each eye was tested at a distance of 25 cm. Color vision testing was performed using Ishihara plates. Contrast sensitivity function testing was evaluated with the vision contrast testing system (VCTS 6000, Vistech Consultants, Inc. at the Research Institute of Ophthalmology in Egypt), The test was done at a distance of 25 cm for each eye separately with the child wearing his optical correction. A quality-of-life questionnaire developed in the department was given to all children and their parents at the initial visit and was given again after 1 month of using the aid; and then again, after 2 months, 6 months and 1 year. The questionnaire contained items related to daily activities of children, like school activities, outdoor activities and other social activities like watching TV and sharing with others during sports and games.

### Prescription of Low Vision Aids

Distance Telescopes: The initial magnification power used for testing was predicted from the ratio of the denominator of the measured visual acuity to the denominator of the desired visual acuity.

The eye with better contrast acuity or visual field was preferentially fitted. Binocular telescopes were used for children who exhibited binocular vision. Also the data collected from the questionnaire was used to guide the prescription of the desired magnification. After predicting the suitable magnification required for the patient, a series of suitable telescopes which gave the desired magnification were put in a suitable frame, with the pupillary inlet coinciding with the visual axis of the patient's eye. The visual acuities of the patient with different telescopes were recorded, and then the suitable one was chosen.

Near Vision Aids: The required starting addition was determined using the pre-calculated magnification values printed in Keeler's chart. The starting addition required for near vision was determined using the Kestenbaum's role (using the reciprocal of the distance visual acuity to calculate the dioptric power of the addition). This addition power was then refined by asking the patient to read a continuous text (school books), and the power was adjusted accordingly. After choosing the appropriate reading aid, the patient's speed of reading was measured to be used as a baseline value to assess the improvement of the child's reading abilities in the following visits.

All children received in-office training sessions to familiarize them with the uses and limitations of the optical systems prescribed until the child demonstrated adequate skill, not necessarily proficiency, in the use of the device before taking it home. Then, the patients were instructed about the methods of care, cleaning and maintenance of the optical device.

The patients were examined after 1 month, 2 months, 6 months and 1 year from the time of finishing their training sessions. During each follow-up visit, the visual function of the patient was evaluated, and the assessment questionnaire was repeated. The patient's performance of different tasks using the aid was discussed with the parents, and all aspects of difficulty in performance were noted and worked upon in the following visit.

Descriptive analysis was used to interpret the results.

## RESULTS

There were 27 males and 23 females with ages ranging from 5 to 15 years, with the mean age ± SD being 11.04 ± 2.579. The age of onset of visual impairment ranged from 1 to 12 years, with the mean age ± SD being 6.44± 2.8078. The duration of visual impairment ranged from 1 to 10 years, with the mean duration ± SD being 4.6± 2.3299. Patients were subjected to complete visual function evaluation. Following a complete clinical examination, fluorescein angiography and electrophysiological tests the following diagnoses were made. Twenty two (44%) patients had familial dominant drusen or other types of hereditary maculopathies, 11 (22%) patients had retinitis pigmentosa, 9 (18%) patients had optic atrophy and 8 (16%) patients had congenital high myopia and other congenital anomalies, namely, microphthalmia and iris and optic disc coloboma. The near visual acuities ranged from A10 to A20, with the mean near acuity ± SD being A13.632± 3.17171. Far visual acuities ranged from 4/60 (0.06) to 6/24 (0.25), with mean distance visual acuity ± SD being 0.122± 0.1191. Interpretation of Ishihara's color plates revealed that 31 (62%) patients were color blind, while 12 (24%) patients had impaired color perception, especially either for red or green; the remaining 7 (14%) patients had normal color prescription. All patients had impaired contrast sensitivity function when tested with the VCTS chart for all spatial frequencies. Testing for contrast sensitivity function demonstrated that for high spatial frequencies (12, 18 cycles/ degree), there were 18 (36%) patients with severe impairment and 5 (10%) patients with mild impairment, while the remaining 27 (54%) patients had moderate impairment of the contrast sensitivity function. The contrast sensitivity function for mid-spatial frequencies (3, 6 cycles/ degree), was severely impaired in 11 (22%) patients, moderately impaired in 36 (72%) patients and mildly impaired in 3 (6%) patients.

Far vision aids were prescribed according to the visual acuity and the visual needs of every patient (Table 1). The most frequently used far vision aid was the 4.2X uniocular spectacle-mounted telescope, which was prescribed for 34 (68%) patients. Two (4%) patients were prescribed the same telescope in a finger ring form. Two (4%) patients were prescribed uniocular spectacle-mounted telescope of power 2.8X. Three (6%) patients were prescribed binocular telescope glasses of power 2.8X. Two (4%) patients were prescribed 6X telescopes in a finger ring form. Two (4%) patients were prescribed 8X telescope in a neck cord form, and 5 (10%) patients were prescribed 1.9X uniocular spectacle-mounted telescope.

**Table 1:** Prescribed far vision aids

Far vision aid	Frequency patients	Percentage
4.2X uniocular spectacle mounted telescope	34	68
4.2X finger ring form	2	4
2.8X uniocular spectacle mounted telescope	2	4
2.8X binocular telescope glasses	3	6
6X telescopes in a finger ring form	2	4
8X telescope in a neck cord form	2	4
1.9X uniocular spectacle mounted telescope	5	10

Regarding near vision aids (Table 2), 8-diopter (D) near reading addition was the most frequently prescribed near vision aid; it was prescribed for 16 (32%) patients, while 16-D illuminated stand magnifiers were prescribed for 8 (16%) patients, 6 (12%) patients were prescribed 12-D binocular reading addition, 5 (10%) patients were prescribed 24-D wide-view reading microscope, 2 (4%) patients were prescribed 16-D reading addition, 8-D hand-held magnifier, 32-D wide-view reading microscope and 16-D illuminated hand-held magnifier together with 16-D near reading addition were prescribed with the same frequency 3 times for each 9 (18%), 2 (4%) patient received 8-D reading addition together with 8-D hand-held magnifier and 2 (4%) patient received 24-D illuminated hand-held magnifier with 16-D near reading addition.

**Table 2:** Prescribed near vision aids

Near vision aid	Frequency patients	Percentage
8-diopter (D) near reading	16	32
16-D illuminated stand magnifiers	8	16
12-D binocular reading addition	6	12
24-D wide-view reading microscope	5	10
16-D reading addition	2	4
8-D hand-held magnifier, 32-D wide-view reading microscope and 16-D illuminated hand-held magnifier together with 16-D near reading addition	9	18
8-D reading addition together with 8-D hand-held magnifier	2	4
24-D illuminated hand-held magnifier with 16-D near reading addition	2	4

Of the patients who received distance vision aids, 5 (10%) patients achieved corrected distance visual acuity of 6/9, 11 (22%) patients achieved aided distance visual acuity of 6/12, 14 (28%) patients achieved aided distance visual acuity of 6/18, 12 (24%) patients achieved aided far visual acuity of 6/24, 6 (12%) patients achieved aided far visual acuity of 6/36, and 2 (4%) patients achieved aided far visual acuity of 6/60.

The effect of near vision aids on the near visual acuity was as follows: 33 (66%) patients achieved aided near visual acuity of A10, 6 (12%) patients achieved aided near visual acuity of A12, 4 (8%) patients achieved aided near visual acuity of A11, 4 (8%) patients achieved aided near visual acuity of A13, and 3 (6%) patients achieved aided near visual acuity of A14.

All patients and parents responded to the questionnaire at the initial visit to assess the degree of impairment of visual performance, and then the questionnaire was administered after 1, 2, 6 months and 1 year. Analysis of the effect of the visual aids in improving the visual performance suggested that the number of patients watching television increased from 18 (36%) to 32 (64%) with the use of the aids; the number of patients who could copy text from the blackboard increased from 4 (8%) to 12 (24%). However, the aids did not appear to have an effect on outdoor and leisure activities in that the number of patients who participated in leisure activities or who could navigate alone did not increase. The ability of the patients to copy from books was improved, as the number of patients who could copy from books increased from 21 (42%) to 37 (74%) with the use of near vision aids. The reading speed improved after using the reading aids in 42 (84%) patients; 24 (48%) of them achieved aided reading speed of more than 60 words per minute (Table 3).

**Table 3:** The Effect on Reading Speed

Reading speed before the aid	Reading speed after the aid			Total

	<20 Wpm	20-60 Wpm	>60 Wpm	
<20 Wpm	8 (32%)	16 (64%)	1 (4%)	25
20-60 Wpm		2 (11.1%)	16 (88.9%)	18
>60 Wpm			7 (100%)	7

Patient compliance with the visual aids was assessed after 1 year of follow-up. Forty four patients remained users of their near vision aids; 38 (76%) of them used the near aid for reading both at home and in the classroom, while 6 (12%) patients used the aid for reading at home only, and the remaining 6 (12%) patients stopped using the aid.

After 1 year of follow-up, 38 (76%) patients were remained users of their aids, while 12 (24%) stopped using it without any influence of the age. Ten (20%) patients were found to be using the far vision aid daily for more than 1 hour per day, while 16 (32%) patients were found to be using the aid daily for less than 1 hour per day; and 10 (20%) patients are still using the aid but not everyday.

## DISCUSSION

In 1992, American Academy of Ophthalmology (AAO) defined low vision as an impairment of visual acuity of less than 6/18 or as restriction of visual field to less than 10 degrees from the point of fixation.^7^ In 1997, American Optometric Association (AOA) added more criteria to the definition of low vision, such as impairment in contrast sensitivity function, color vision and ocular motility.^8^

The aim of our work was to assess the different aspects of visual function impairment in children with low vision and to evaluate the role of visual aids in improving their visual performance and in keeping them socially as well as educationally integrated.

Our study showed that all children with low vision had impaired contrast sensitivity function for all spatial frequencies; and in particular for mid-spatial frequencies, which are considered the accurate indicators for visual performance. Patients with severely impaired contrast sensitivity function showed lower reading speeds than those with the same near visual acuity but better contrast sensitivity function.^9^

Our results are in agreement with those reported in other studies, wherein it was concluded that many patients respond well to low vision aids while others do not. These differences may be due to the variations in contrast sensitivity function, and so appropriate diagnostic use of contrast sensitivity function can explain the failure of low vision aids in some patients.^10^ It was found that reading performance and reading rate are affected greatly by the contrast sensitivity function of the patients.^11^ In addition, contrast sensitivity, especially in low and medium spatial frequencies, is a better indicator for reading performance as compared with visual acuity. It was also found that even a small reduction in contrast reduced reading performance in almost all low vision patients tested, hence the need for higher magnification for reading.^12^

On the other hand, no significant correlation between contrast sensitivity and reading performance in children was found.^5^ After investigating 71 children with low vision, it was noted that visual performance is affected mainly by the child's age. It was reported that contrast sensitivity in normal children did not reach adult levels till the age of 7 or 10 years and therefore should not be used in the assessment of visual performance in the pediatric age group. Success in this study was assessed by the improvement in visual acuity of more than 6/24 for distant vision and A12 for near vision, the improvement in visual performance both in classroom and in social activities, and the patient's compliance with the aid.^13^

In regard to total for visual acuity improvement, 42 (84%) patients could achieve aided visual acuity of 6/24-6/9. These results matched the results of two previous studies concerning the efficiency of low vision aids in improving visual acuity. They studied visual rehabilitation for 96 patients of different age groups with advanced stages of glaucoma, optic atrophy, myopia and retinitis pigmentosa and found that 100% of patients showed improvement in visual acuity for both far vision and near vision with the use of low vision aids.^14^ Another study evaluated the effect of low vision aids on visual acuity of 71 patients with fundus flavimaculatus and Stargardt's maculopathy and found that in all of them low vision aids improved visual acuity for both distant vision and near vision.^15^

In this study improvement in near vision was seen in 43 (86%) patients who after receiving visual aids could read the print size of school books (A12-A10). These results are in accordance with previous work the showed that low vision aids were very effective in helping 90% of the low vision patients to read normal size prints.^16^

Distance telescopes in this study were helpful in allowing children to function independently especially at school. It was also found that telescopes had no effect on mobility performance, as there was no remarkable increase in the number of patients who could walk alone or share during sports and games with the use of the telescope. These results matched the data obtained in a survey that assessed the user success for distance telescopes in 142 patients using various types of telescopes and found that telescopes were effective in improving visual performance both outdoors and indoors.^17^ Also another study that assessed low vision rehabilitation of 220 children found that a large number of school children depended on distance telescopes for visualization in the classroom.^18^

Patient's compliance with the telescopic aid, after 1 year, in this report was similar to that achieved by Lowe and Rubinstein,^17^ who studied the user success for distance telescopes in 142 individuals of different age groups and with various causes of low vision; and they found that 133 (93.5%) individuals remained users of their telescopes with the same frequency outdoors and indoors. Also, low vision rehabilitation was studied in 220 children, and it was found that a large number of school children used distance telescopes in classroom.^18^ In another study, the effect of low vision aids was assessed among preschool children; and after 2 years, it was found that 50% of the children remained users of both far vision and near vision aids, with improvement of visual function. The lower degree of compliance in this study may have been, related to the lack of interest in reading and visualizing far objects in pre-school children.^19^ A limitation of this study was the use of a non validated questionnaire to obtain feedback.

To summarize, low vision is a problem which has a wide-ranging impact on the behavior of children and adolescents in the social and educational spheres of life. Vision rehabilitation with the use of optical vision aids was found to be very helpful in minimizing the impact of low vision and in improving daily performance of the visually impaired patients.
